# Comment on Beyond the guidelines: real-world challenges in rabies post-exposure prophylaxis

**DOI:** 10.1093/jtm/taag012

**Published:** 2026-02-03

**Authors:** Guillermo Mena, Marta Aldea

**Affiliations:** Department of Preventive Medicine and Epidemiology, Hospital Clínic Barcelona, c/Rosselló 138, 08036, Barcelona, Spain; Faculty of Medicine, University of Barcelona, c/ Casanova 143, 08036, Barcelona, Spain; Department of Preventive Medicine and Epidemiology, Hospital Clínic Barcelona, c/Rosselló 138, 08036, Barcelona, Spain; Faculty of Medicine, University of Barcelona, c/ Casanova 143, 08036, Barcelona, Spain

**Keywords:** rabies, vaccination, pep, immunoglobulin, postexposure, prophylaxis

We are writing in response to the article by Díaz-Menéndez and colleagues,^1^ and we thank the authors for their valuable insights.

We agree with their conclusion that current WHO guidelines lack the granularity required to navigate less typical exposure scenarios. Our clinical team also concurs with the exposure classification applied in all three cases described in the article. Given the theoretical risks outlined, we would likewise have adopted a maximal approach, managing all three situations as WHO Category III exposures.

We would like to comment specifically on the management of Case 3, involving a 36-year-old nurse who manipulated a dog-bite wound later associated with fatal rabies, without gloves and in the presence of potential periungual skin disruption due to habitual nail-biting. In this case, rabies vaccination was administered as a precaution, while rabies immunoglobulin (RIG) was not given because several months had elapsed since the exposure.

In our view, post-exposure prophylaxis in this scenario should have included both a full vaccination schedule and RIG. According to WHO recommendations, Category III exposures warrant the administration of RIG, except in immunocompetent individuals with a correctly documented prior rabies vaccination.^2^

Furthermore, prolonged incubation periods exceeding 1 year have been documented, including a fatal bat-associated rabies case occurring more than 6 years after exposure.^3^ The most recent WHO position paper states that the likelihood of developing clinical rabies declines progressively during the 12 months following exposure, with clinical disease occurring only rarely thereafter, and that vaccination may be reserved for exposures within this period when vaccine supply is limited.

In conclusion, we propose that, following individualized risk assessment and whenever RIG is available, full post-exposure prophylaxis should be administered, particularly when the interval since exposure does not exceed 1 year. Moreover, in cases involving contact with body fluids known to be infected with rabies virus, such as in Case 3, we believe that full post-exposure prophylaxis should be considered even when more than 1 year has elapsed since exposure.
